# A naturalistic study of yoga, meditation, self-perceived stress, self-compassion, and mindfulness in college students

**DOI:** 10.1080/21642850.2019.1688154

**Published:** 2019-11-18

**Authors:** Margaret M. Gorvine, Nickolas D. Zaller, Heather K. Hudson, Denise Demers, Lyndsay A. Kennedy

**Affiliations:** a Department of Health Behavior and Health Education, Fay W. Boozman College of Public Health, University of Arkansas for Medical Sciences, Little Rock, AR, USA; b Department of Health Sciences, University of Central Arkansas, Conway, AR, USA; c Department of Psychology, Hendrix College, Conway, AR, USA

**Keywords:** Yoga, mindfulness, meditation, mind–body interventions, self-compassion, stress

## Abstract

**Objective:** This study compared the effects of yoga and mindfulness meditation on self-compassion, mindfulness, and perceived stress in college students; and explored mind–body mechanisms and predictors of stress reduction.

**Participants:** Student participants (*N* = 92) were enrolled in either yoga or mindfulness meditation classes at a college in the southern United States from August through May of 2015–2016.

**Methods:** Students participated in 50-minute classes twice a week for 10 weeks, completing self-report questionnaires during the 1st and 10th week.

**Results:** Multiple-linear regression analysis found change in self-compassion was the strongest predictor of stress reduction.

**Conclusions:** Increasing self-compassion may increase the efficacy of mind–body interventions. Research into mind–body mechanisms is needed to identify intervention components that most improve student well-being.

Stress on college campuses is significant; 82% of students report feeling overwhelmed by their responsibilities and 60% report higher than normal or tremendous levels of stress (American College Health Association, 2013). College administrators are tasked with addressing myriad student health and wellness needs – often with limited resources. Therefore, research into preventive mind–body interventions that offer low-cost and effective services to reduce negative health outcomes among college students ought to be prioritized.

One factor that may be promising to target in preventive mind–body interventions is self-compassion. Neff defines self-compassion as including three sub-constructs: ‘(a) self-kindness – being kind and understanding toward oneself in instances of pain or failure rather than being harshly self-critical, (b) common humanity – perceiving one’s experiences as part of the larger human experience rather than seeing them as separating and isolating, and (c) mindfulness – holding painful thoughts and feelings in balanced awareness rather than over-identifying with them’ (Neff, 2003, p. 85). Importantly, research has found that self-compassion positively correlates with health behaviors and well-being (Baer et al., 2013; Murphy, Mermelstein, Edwards, & Gidycz, 2012). Similar results have been found with mindfulness. Brown and Ryan define mindfulness as ‘being focused on the presence or absence of attention to and awareness of what is occurring in the present’ (Brown & Ryan, 2003). Mindfulness has been positively associated with health and increased energy level in college students (Bodenlos, Noonan, & Wells, 2013). For example, researchers studied one published mindfulness-based protocol, Koru, developed specifically for college students by psychiatrists at Duke University (Rogers & Maytan, 2012) and found significant increases in both self-compassion and mindfulness, as well as improved sleep (Greeson et al., 2011) after participating in the mindfulness meditation intervention. Research on brief formats of Mindfulness-based Stress Reduction (MBSR) (Kabat-Zinn, 1984) – the flagship of mindfulness-based interventions – indicates that low dose interventions may be as effective in increasing self-compassion and mindfulness (Bergen-Cico, Possemato, & Cheon, 2013).

Additionally, both yoga and mindfulness interventions have been shown to reduce self-reported stress and biomarkers of stress (Falsafi & Leopard, 2015; Melville, Chang, Colagiuri, Marshall, & Cheema, 2012; O’Driscoll, Byrne, Mc Gillicuddy, Lambert, & Sahm, 2017; Regehr, Glancy, & Pitts, 2013; Sivasankaran et al., 2006). Other research has shown that yoga increases mindfulness (Shelov, Suchday, & Friedberg, 2009), and self-compassion in some cases (Toise et al., 2014). In contrast, in a recent controlled trial, a mindfulness intervention positively impacted self-compassion scores, while the yoga intervention did not (Falsafi, 2016). This points to inconsistency in the evidence that requires further exploration.

A better understanding of the effectiveness of mind–body interventions and the ‘active ingredients’ in yoga and meditation interventions is also needed to be able to make informative comparisons. Previous research comparing yoga and mindfulness as well as one mechanism (heart rate variability – a physiological marker of stress reduction) supports the efficacy of a combined yoga and mindfulness intervention for college students (Hunt, Al-Braiki, Dailey, Russell, & Simon, 2018). Specifically, continued evaluation of the best predictors to bolster within an intervention, such as self-compassion and mindfulness, to decrease stress and potentially increase health-promoting behaviors will aid in future intervention development for increasing student well-being.

Further exploration of MBSR adaptations and mindfulness-based interventions for the college student population is important in order to better understand the mechanisms of change. Therefore, this study examined two mind–body interventions: yoga and mindfulness meditation in natural class settings at a small liberal arts college in the southern United States. Specifically, this study explored and compared the effects of 20 classes of either yoga, which included instruction of beginning standing poses (*āsanas*), breathing exercises (*prāṇāyāma*) as well as relaxation (*śavāsana*), to 20 classes of mindfulness meditation, which included sitting meditation, walking meditation, and body scan (all three of which are used in the MBSR curriculum).

The first aim of this research was to compare the effectiveness of the yoga and meditation classes on reducing stress, as well as on increasing self-compassion and mindfulness. The secondary purpose was to compare two possible ‘active ingredients’ in these interventions – mindfulness and self-compassion – in predicting the reduction of students’ self-perceived stress. The study evaluated existing wellness classes and did not employ an active control due to constraint of resources, which limits the intervention effect data. Nonetheless, the results add to the understanding of the mechanisms of mind–body interventions.

## Methods

### Participants

The participants (*N* = 92) for this study were undergraduate students at a small liberal arts college in the southern United States during the 2015–2016 academic year. As part of the graduation requirements, students at this college select two for-credit activity classes some time during their four years at the college. The majority of the for-credit activity classes are exercise-based. The students for this study registered for one of two yoga classes, offered at 7 am or 8 am or the mindfulness meditation class that was held at 11:00 am during fall of 2015 and spring of 2016.

Individuals in the activity classes described above were invited to participate in the study by filling out questionnaires. Student participation in the study was entirely voluntary and no identifying information was collected. If interested in participating, students received a brief explanation of the study, underwent informed consent, and signed informed consent forms that were kept separate from the participants’ unidentified questionnaires. Part of the consent process included highlighting that participation would not affect the students’ grades; grades were based solely on attendance and students received pass or fail (credit or no credit).

**Figure 1.**
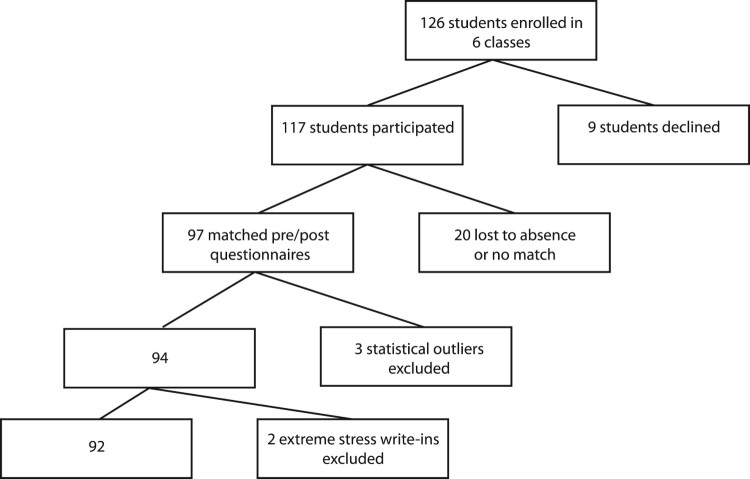
Study flow chart.

The total number of students that enrolled in the fall and spring semesters for all of the classes was 126. Of those, 117 completed at least one survey. Twenty pre- and post-questionnaire matches were not included either because of absence on the day of surveying or because the participant did not provide the same 4 digit code to link the pre and post forms. In total, there were 97 matching pairs of completed (pre and post) questionnaires collected. Three more students’ data were excluded during the data analysis as they contained statistical outliers in self-compassion, mindfulness, stress, or a combination of all three. Because two students wrote in (unprompted) details of highly stressful adverse life events, it was decided their data were not representative of the general student population, therefore their data were excluded. The study flow chart is depicted in Figure 1.

Prior to the data collection, the researcher received approval in March and April of 2015 from two Institutional Review Boards – one from the researcher’s Master’s study university, and the second from the liberal arts college where the interventions/classes were held and data were collected. Informed consent was obtained from all individual participants included in the study.

### Study site

During the 2014–2015 academic year, the institution under study had an undergraduate student enrollment of 1,338 with 99% of undergraduate students considered full-time. Males represented less than half (46.9%) of the undergraduate population. Racial/ethnic demographics included: 77.2% White, non-Hispanic; 4.8% Black, non-Hispanic; 4.9% Hispanic; 4.5% Asian; 2.7% selected two or more races; 0.5% American Indian or Alaska Native; and 0.1% Native Hawaiian or Pacific Islander; and 4.4% non-resident alien (National Center for Education Statistics, 2015). The institution under study provided four-year liberal arts education.

### Measures

The study used three scales and an additional questionnaire to collect more specific data pertaining to health behavior and demographics.

#### Perceived stress scale 10 (PSS-10)

To assess the participants’ level of self-perceived stress, this study employed the Perceived Stress Scale 10 (PSS-10) (Cohen, Kamarck, & Mermelstein, 1983), which has been widely used in social psychological research for three decades. The PSS-10 questionnaire aims to capture the participants’ stress levels via questions such as: ‘In the last month, how often have you felt nervous and ‘stressed’?.’ In a study with 510 college-student participants, the scale’s coefficient alphas in the three samples were .84, .85 and .86 and the test-retest reliability score was .85 (Cohen, Williamson, Cohen, & Williamson, 1988).

The PSS-10 scale included 10 items, with the following response values: 1 = Never, 2 = Almost Never, 3 = Sometimes, 4 = Fairly Often, 5 = Very Often. The self-perceived stress scores were tabulated by reversing the scores on the four positive items and then summing scores for all 10 items. The higher the score, the higher the level of self-perceived stress.

#### Mindful awareness attention scale (MAAS)

In order to study mindfulness as conceptualized by Brown and Ryan (2003), we used the Mindful Awareness Attention Scale (MAAS) (Brown & Ryan, 2003). The MAAS asks questions to assess levels of mindfulness in everyday life. An example question is: ‘I find it difficult to stay focused on what’s happening in the present.’ This scale has demonstrated good internal validity (α = .82) (Brown & Ryan, 2003). In order to align with the other scales in the survey, the scores were reverse coded so that higher scores indicated higher levels of mindfulness.

#### Self-compassion scale – short form (SCS-SF)

Levels of self-compassion were measured using the Self-Compassion Scale – Short Form (SCS-SF) (Raes, Pommier, Neff, & Van Gucht, 2011). Three constructs of self-compassion were measured including: self-kindness, common humanity, and mindfulness (Neff, 2003). An example question is: ‘I try to be understanding and patient towards those aspects of my personality I don’t like’ (Neff, 2003, p. 230). The long form of the scale has demonstrated good internal validity (α = .82), while the short form has shown almost perfect correlation to the long (validated) form, *r* ≥ 0.97 (Raes et al., 2011). The higher the SCS-SF score the higher the level of self-compassion.

### Data analysis

A one-way between groups analysis of covariance (ANCOVA) measured the difference of effect of each intervention on stress, self-compassion, and mindfulness while controlling for pre-intervention differences. Additional data analysis included matched pair *t* tests to determine the effect of the interventions on mindfulness, self-compassion, and self-perceived stress. Multiple-linear regressions examined the predictive value of self-compassion and mindfulness on stress reduction in each of the interventions.

This was a pilot study and therefore sample size considerations were based largely on feasibility. We estimated our sample size based on potential effect sizes outlined by Birnie, Speca, and Carlson (2010) and Greeson, Juberg, Maytan, James, and Rogers (2014) and power analyses were conducted via the G*Power program (Faul, Erdfelder, Lang, & Buchner, 2007). Our power analyses determined a required sample size of *N* = 23 to detect effect sizes of d = .45 or greater, with 80% power and an alpha level of 0.05. We acknowledge that our final sample was insufficiently powered to detect smaller effect sizes between groups.

### Procedure

The yoga and meditation classes were held in the movement studio on campus, which is well-equipped with props to make the participants comfortable in their classes including blocks, straps, and mats. The instructor (who was also the researcher for this study) held Experienced Registered Yoga Teacher 200 (E-RYT) registration through Yoga Alliance, which mandates 200 h teacher training and over 1000 h of teaching experience. In addition to *Haṭha* yoga training, the instructor had practiced meditation for 25 years, and had attended the 7-Day Mindfulness-Based Stress Reduction (MBSR) Professional Training (Santorelli & Kabat-Zinn, 2014) with Jon Kabat-Zinn and Saki Santorelli. The instructor had taught movement and meditation since 1999 and *Haṭha* yoga since 2003. The semesters studied were the instructor’s eighth and ninth semesters teaching at the college.

The initial recruitment, enrollment and data collection began the second week of the Fall 2015 semester, and the second week of the Spring 2016 semester, respectively. During the eleventh week of class during both semesters, follow-up surveys were administered. At the bottom of each survey, a class code identified which class the survey belonged to. The first two sections of the survey (PSS-10 and SCS-SF scales) were completed on Scantron^©^ forms. The MAAS scale was filled out on paper. The participants chose a 4-digit code that they wrote at the top of the hand-written pages and the Scantron^©^ form. The participants stapled the Scantron^©^ form and the questionnaires together and placed them in the slot of a covered box.

#### Yoga intervention

A description of the postures (*āsanas*) practiced, and the approximate timing of the sections were recorded as field notes by the researcher directly after the first 7 am class (between 7:50 am and 8:00 am) and then the same teaching sequence was repeated with the following 8 am class during the fall semester. Each of the poses and activities performed in the 7 am class were approximated in the 8 am class as closely as possible even to the extent of anecdotal stories, and relaxation (*śavāsana*) instructions. Days when a new pose was introduced, the flow stopped for a few minutes to explain details about the alignment. The majority of the classes included music. The 50-minute class time included taking attendance, as well as cleaning and returning equipment to storage. The format of the class usually included 10 min of warming up, 15–20 min of standing poses with optional forms of *vinyāsas* (which usually include four connecting poses of plank, ‘yoga push-up’ (*caturaṅga daṇḍāsana*), up dog (*urdhva* mukha śvānāsana), and down dog (*adho mukha śvānāsana*), but can be performed with knees down instead of in plank position, for example) in between standing poses, 5 min cooling/stretching, and 10 min guided relaxation/meditation in corpse pose (*śavāsana*).

#### Meditation intervention

Soft exercise mats, chairs, and blocks aided the mindfulness meditation classes – which helped the students find their most comfortable sitting meditation positions. Activities in addition to the sitting meditation included walking meditation and body scan exercises, which includes a guided ‘tour’ bringing one’s attention to each part of the body in a slow, sequential process. The meditation classes began with a short introduction about meditation, including topics in the 7-day MBSR Professional Training materials (Santorelli & Kabat-Zinn, 2014), such as non-judgment, patience, beginner’s mind, trust, letting go, and non-striving, or instruction about new techniques such as body scan and walking meditation. The meditation classes included a combination of up to 20 min of sitting mindfulness meditation interspersed with 10 min of walking meditation. Body scans lasted up to 30 min.

#### Ethics Statement

All procedures performed in studies involving human participants were in accordance with the ethical standards of the institutional and/or national research committee and with the 1964 Helsinki declaration and its later amendments or comparable ethical standards.

Informed consent: Informed consent was obtained from all individual participants included in the study.

## Results

The participants consisted of 31.5% male (*n *= 29) and 67.4% female (*n *= 62), and one participant did not identify their sex. The mean age of the participants was 20.18 years old. The participants’ racial/ethnic demographics were not collected as the lack of diversity within each class would compromise the students’ anonymity.

### Ancova

A one-way between groups analysis of covariance was conducted to compare the effectiveness of the two different interventions (yoga and meditation) on reducing students’ level of self-perceived stress. The independent variable was the type of intervention (yoga vs. meditation), and the dependent variable consisted of the post scores on the PSS-10. Participants’ pre-intervention PSS-10 scores were used as the covariate in this analysis.

Initial checks were conducted to ensure that there was no violation of the assumptions of normality, linearity, homogeneity of variances, homogeneity of regression slopes and reliable measurement of the covariate. After adjusting for the pre-intervention scores, there was no significant difference between the two intervention groups on the PSS-10 scores (*p *= .38).

Similarly-structured one-way between groups analyses of covariance were run to compare the effectiveness of the two different interventions (yoga vs. meditation) on increasing students’ self-compassion (*p* = .18) and mindfulness (*p* = .45). The groups’ pre-intervention scores for self-compassion or mindfulness did not differ significantly. No significant difference was found between the intervention groups.

In addition, a two-way analysis of covariance found no statistical significance or difference in the effect of the interventions on reducing stress for males versus females, despite a statistically significant difference in the pre-intervention stress scores for males (*M *= 26.68, *SD* = 6.62) who scored lower on the scale than females (*M *= 29.74, *SD* = 6.78, *p *< .05).

### T-tests

Additionally, separate repeated measures *t* tests for each condition were run to determine the effect of each intervention on pre- and post-intervention stress, mindfulness, and self-compassion. The separate *t* tests for the yoga and meditation conditions did not show significant stress reduction, but did demonstrate small to medium effect sizes in each condition for increased self-compassion and mindfulness.

As no between group difference was found in the ANCOVA analysis for stress, the data for both interventions were combined and *t* tests were conducted to measure the changes in mindfulness and self-compassion pre- and post-intervention in both groups combined. The combined mind–body group had small effect sizes in decreasing self-perceived stress and increasing self-compassion and mindfulness. The results of the data analysis of matched pair *t* tests to determine the overall effect of the combined mind–body intervention group on mindfulness and self-compassion, as well as self-perceived stress are listed in Table 1.

**Table 1.** Paired samples statistics: stress, self-compassion, and mindfulness in combined group (yoga & meditation).

	Pre		Post		d*df*	*t*	*p*	Cohen’s *d*
	M	SD	M	SD				
Stress	28.77	6.88	27.09	6.71	89	2.35	.021	.25
Self-Compassion	34.74	8.66	38.06	6.55	86	−5.23	.000	−.43
Mindfulness	56.05	12.32	58.87	12.7	90	−3.05	.003	−.23

### Multiple-linear regression

To answer the research question, ‘Which variable, self-compassion or mindfulness, is more predictive of stress reduction after each intervention?’ standard multiple-linear regressions were calculated using the change scores for self-compassion and mindfulness to predict post stress scores.

A hierarchal model included pre-intervention stress scores as the first covariate, and change in self-compassion and change in mindfulness as second level covariates for the dependent variable, post-stress.

For the yoga intervention, pre-intervention stress scores explained 19.8 percent of the variance in the change of perceived stress. For the other predictors, only the change in self-compassion scores was statistically significant. The full results of this analysis are presented in Table 2.

**Table 2.** Multiple linear regression: change in self-perceived stress in yoga intervention.

	*df*	*F*	*R^2^*	*β*	*p*
Pre-intervention Stress	45	10.88	.198	.445	.002
Δ Self-Compassion				−.285	.042
Δ Mindfulness				−.237	.087

For the meditation intervention, pre-intervention stress scores explained 30.1 percent of the variance in the change of perceived stress. Of the other predictors, only the change in self-compassion scores was statistically significant. The full results of this analysis are presented in Table 3.

**Table 3.** Multiple linear regression: change in self-perceived stress in meditation intervention.

	***df***	***F***	***R^2^***	***β***	***p***
Pre-intervention Stress	39	16.397	.301	.651	.000
Δ Self-Compassion				−.472	.000
Δ Mindfulness				−.044	.709

## Discussion

The results reveal insights for mind–body intervention research and that could inform program design. Among the most interesting results was the strength of self-compassion as a predictor for stress reduction in both intervention conditions – the stronger of which was evident in the meditation condition. In contrast, mindfulness was not a statistically significant predictor in either condition. Although it should be noted that there was no difference between groups in the development of self-compassion, the strong influence of self-compassion on predicting stress reduction could have resulted from the additional time spent in the meditation class talking about developing a compassionate stance toward self, as compared to more time spent focusing on body position in the yoga condition.

In addition, the increase in self-compassion as a predictor for stress reduction across both intervention conditions (yoga and meditation) adds to the evidence for including self-compassion training (either directly or indirectly) in programming designed to improve student well-being. The lack of difference between meditation and yoga on reducing stress bolsters evidence for including both types of classes on college campuses – allowing students the choice of how to pursue their well-being. This study also points to the opportunity for the development of more targeted interventions that address self-compassion and mindfulness for college students. In addition, yoga instruction that intentionally incorporates developing self-compassion may prove more effective at reducing student stress. Regardless, student wellness programming that includes augmenting self-compassion and mindfulness can be developed and delivered in existing activities courses with minimal investment and maximum impact.

### Limitations

While this study design was quasi-experimental and was conducted in a naturalistic setting, these limitations can be also seen as strengths in that the design directly compared two classes that may be available at similar institutions. Nonetheless, the design lacked a control group and was not randomized as the participants were drawn from a convenience sample of students who were already enrolled in the yoga and meditation classes, which increased the likelihood of self-selection bias. In addition, the primary investigator was also the yoga and meditation instructor. Though having the same instructor across conditions provides important consistencies, it also opens up the possibility of unconscious bias from the researcher influencing the results. In addition, collecting self-report data without biological markers of stress limits the accuracy of the stress measurements.

## Conclusion

This study provides preliminary evidence that yoga and meditation classes increase both self-compassion and mindfulness, and may help to reduce stress among college students. When compared to each other, the yoga and meditation classes were not found to have statistically significant different effects in reducing stress or increasing self-compassion or mindfulness. This study also presents preliminary evidence that self-compassion predicts stress reduction in mind–body interventions. Therefore, targeting self-compassion in wellness programming for college students may increase the efficacy of mind–body interventions and ought to be considered in future curricula. Further research into the mechanisms of mind–body interventions will help identify the components that most improve student well-being.
